# Alteration of gut microbiota in high‐fat diet‐induced obese mice using carnosic acid from rosemary

**DOI:** 10.1002/fsn3.2841

**Published:** 2022-05-24

**Authors:** Xuan He, Man Zhang, Shu‐Ting Li, Xinyu Li, Qingrong Huang, Kun Zhang, Xi Zheng, Xue‐Tao Xu, Deng‐Gao Zhao, Yan‐Yan Ma

**Affiliations:** ^1^ 47892 School of Biotechnology and Health Sciences Wuyi University Jiangmen China; ^2^ 242612 Department of Food Science Rutgers University New Brunswick New Jersey USA; ^3^ International Healthcare Innovation Institute (Jiangmen) Jiangmen China

**Keywords:** antiobesity, carnosic acid, gut microbiota, rosemary

## Abstract

*Rosmarinus officinalis* (rosemary) is widely used as a food ingredient. Rosemary extract (containing 40% carnosic acid) exhibited potent antiobesity activity. However, the relationship between carnosic acid (CA) and changes in the gut microbiota of high‐fat diet (HFD)‐induced obese mice has not been fully investigated. C57BL/6 mice were fed a normal diet, an HFD, or an HFD containing 0.1% or 0.2% CA for 10 weeks. CA exhibited promising antiobesity effects and caused marked alterations in the gut microbiota of HFD‐induced obese mice. CA caused the prevalence of probiotics and functional bacteria, including *Akkermansia muciniphila*, *Muribaculaceae unclassified*, and *Clostridium innocuum* group, and inhibited diabetes‐sensitive bacteria, including *Proteobacteria* and *Firmicutes*. The ratio of *Firmicutes* to *Bacteroidetes* was regulated by CA in a dose‐dependent manner, decreasing it from 13.22% to 2.42%. Additionally, CA reduced bile acid‐metabolizing bacteria, such as *Bilophila*, *Clostridium*, *Lactobacillus*, and *Leuconostoc*. The results of the linear discriminant analysis and effect size analysis indicated that CA attenuated the microbial changes caused by HFD. The high CA (HCA) group (HFD containing 0.2% CA) exhibited a greater abundance of *Verrucomicrobiae* (including *Akkermansia muciniphila*, genus *Akkermansia*, family *Akkermansiaceae*, and order *Verrucomicrobiales*), *Eubacterium*, and *Erysipelatoclostridium*, and the low CA (LCA) group (HFD containing 0.1% CA) exhibited a greater abundance of *Eisenbergiella*, *Intestinimonas*, and *Ruminococcaceae*. Our results demonstrate that the antiobesity effects of CA might be strongly related to its prebiotic effects.

## Graphical abstract

Carnosic acid caused the prevalence of *Verrucomicrobia* and *Bacteroidetes*. Carnosic acid inhibited *Firmicutes* and *Proteobacteria* and reduced bile acid‐metabolizing bacteria. *Akkermansia muciniphila* is crucial for the antiobesity effect of carnosic acid.

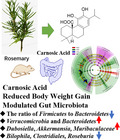

## 1 INTRODUCTION

Obesity is closely linked to several chronic diseases, including cardiovascular diseases, hypertension, and diabetes (Derosa & Maffioli, 2012). Functional food‐based dietary intervention is an effective strategy for the prevention and control of obesity (Fabricatore and Wadden, 2003; Riccardi et al., 2005). Therefore, it is necessary to identify diet‐derived antiobesity compounds with excellent bioefficacy and long‐term safety (Liu et al., 2020).

*Rosmarinus officinalis* L. (rosemary), a widely used food ingredient, is used in folk medicine for the treatment of several disorders, including stomach problems and inflammatory and respiratory symptoms (al‐Sereiti et al., 1999; del Pilar Sánchez‐Camargo & Herrero, ). Previous studies revealed that rosemary extract can limit weight gain and prevent lipid accumulation in hepatocytes by activating the AMPK/PPAR and EGFR/MAPK pathways (Wang et al., 2012; Zhao et al., 2015). The gut microbiota plays an important role in the bioactivity of many natural products (Cotillard et al., 2013). For example, the antidepressant effects of rosemary extract are mediated by rebalancing the gut microbiota (Guo et al., ). Carnosic acid (CA) is the major active component of rosemary and has been extensively studied (Chen et al., 2020). It has been reported that rosemary extracts (containing 40% CA) change the microbiota composition of female Zucker rats, but only a few members of the phyla *Firmicutes*, *Bacteroidetes*, and *Actinobacteria* were studied (Romo‐Vaquero et al., 2014). Thus, the modulatory effect of CA on gut microbiota needs to be investigated further.

C57BL/6 mice were fed an HFD supplemented with or without CA for 10 weeks. To our knowledge, this is the first report on the modulating effect of CA on gut microbiota in HFD‐induced obese mice.

## 2 MATERIALS AND METHODS

### 2.1 General experimental procedures

Sephadex LH‐20 (Amersham Pharmacia Biotech) and D101 macroporous resin (Sinopharm Chemical Reagent Co., Ltd.) were used for column chromatography (CC).

### 2.2 Plant material

The leaves of *R. officinalis* were collected from Yulin (Guangxi, China) in August 2015 and authenticated by Professor Yin Li, School of Pharmacy, Southwest Minzu University, China. A voucher specimen (ID 20150803) was deposited in the herbarium of Materia Medica, Wuyi University, Jiangmen, China.

### 2.3 High‐performance liquid chromatography (HPLC)

A Waters H‐Class instrument (Waters) was used for HPLC analysis. Chromatographic separation was performed on a Hypersil GOLD column (1.9 μm, 100 mm × 2.1 mm) using 0.1% formic acid solution and acetonitrile as the mobile phase.

### 2.4 Extraction and isolation

Dried leaves of *R. officinalis* (12.0 kg) were percolated with ethanol (3 × 25 L) to obtain a crude extract. The extract (561.5 g) was partitioned into water (2.5 L) and extracted successively with hexane, ethyl acetate, and *n*‐butanol. The hexane fraction was subjected to repeated CC to obtain carnosic, carnosol, and rosmarinic acids. Their structures were identified using mass spectrometry and nuclear magnetic resonance spectroscopy. The purities of CA, carnosol, and rosmarinic acid were determined using an ACQUITY UPLC H‐Class system (Waters Co.) with a C‐18 column (Thermo, 1.9 μm, 4.6 mm × 150 mm), and were found to be ≥95%.

### 2.5 Animals

Six‐week‐old male C57BL/6J mice were obtained from GDMLAC. The protocol and experimental procedures for this study were approved by the Ethics Committee for Animal Experimentation of Wuyi University, and they followed the National Institute of Health's Guide for the Care and Use of Laboratory Animals.

The mice were divided into the following groups (*n* = 6 for each group): HFD group (fed with HFD containing 60% calories from fat), low CA (LCA) group (fed with HFD containing 0.1% CA), high CA (HCA) group (fed with HFD containing 0.2% CA), carnosol (CO) group (fed with HFD containing 0.2% carnosol), rosmarinic acid (RA) group (fed with HFD containing 0.2% rosmarinic acid), and the normal diet (ND) group (fed with normal diet containing 10% fat calories). A high‐fat diet (product number: TP23300) and a normal diet (product number: TP233020) were purchased from Trophic Animal Feed High‐Tech Co. Ltd. Food consumption was monitored daily and body weight was measured weekly. The mice were housed under a light/dark cycle (12/12 h) at an ambient temperature of 22 ± 2°C with constant humidity and given water and food ad libitum. Feces were collected three times a week and stored at a temperature of 80°C in a freezer. After 10 weeks, all the mice were sacrificed and the adipose tissue (epididymal, retroperitoneal, and mesenteric), liver, kidney, and spleen were removed, weighed, placed in vials immediately, and frozen in liquid nitrogen.

### 2.6 DNA extraction and 16S rRNA gene sequence analysis

The methods for DNA extraction, sequencing, and data analysis were as described previously by Segata et al. (Segata et al., 2011). Briefly, total DNA was extracted using an E.Z.N.A.^®^ Stool DNA kit (D4015, Omega, Inc.), according to the manufacturer's instructions. Amplicon sequencing was performed using the Illumina MiSeq platform. After merging paired‐end reads (FLASH) and quality control (fqtrim, V0.94), sequences with ≥97% similarity were regarded as the same operational taxonomic units (OTUs) using Vsearch (v2.3.4). The OTUs were classified using the Ribosomal Database Project software, and OTU abundance data were normalized with a standard sequence number. The analyses were performed by LC‐Bio Tech Co., Ltd.

### 2.7 Statistical analysis

Experimental data are expressed as the mean ± standard deviation. The statistical significance was calculated using a one‐way analysis of variance (ANOVA) followed by a post hoc test. Linear discriminant analysis effect size (LEfSe) analysis was performed using the LEfSe software (http://huttenhower.sph.harvard.edu/galaxy/) with a logarithmic discriminant analysis (LDA) threshold score of 4.0.

## 3 RESULTS

### 3.1 Extraction and isolation of compounds from rosemary

**FIGURE 1:**
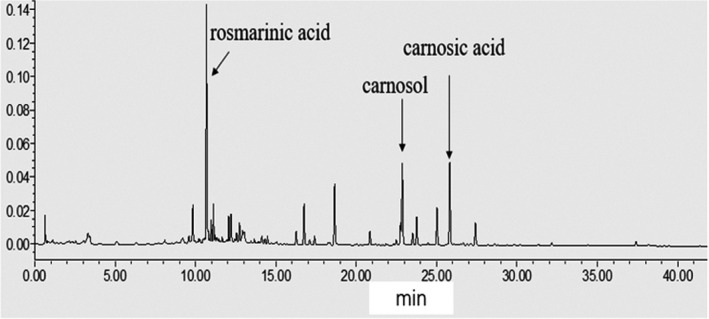
HPLC traces of rosemary extracts

Rosemary is rich in bioactive phenolics, and HPLC analysis has shown that the major bioactive phenols in rosemary comprise of CA (6.30 mg/g), carnosol (3.61 mg/g), and rosmarinic acid (1.92 mg/g) of the dry weight of rosemary (Figure 1, Table 1). The ethanol extract of rosemary was subjected to repeated CC to yield 12 g CA, 5 g carnosol, and 4 g rosmarinic acid.

**TABLE 1:** Validation parameters for HPLC quantitation method of carnosic acid, carnosol, and rosmarinic acid in rosemary

Compound	Concentration (mg/g of dry weight)	Calibration curve	Linear Range (μg/ml)	*r*	LOQ (μg/ml)	LOD (μg/ml)
Carnosic acid	6.30	*y* = 0.12285*x* − 0.0562	0.5–1000	.9997	0.2	0.05
Carnosol	3.61	*y* = 0.18543*x* + 0.07234	0.5–1000	.9994	0.2	0.05
Rosmarinic acid	1.92	*y* = 0.88664*x* − 0.19306	0.5–1000	.9999	0.5	0.2

### 3.2 Carnosic acid reduced body weight gain and food efficiency

Previous studies found that rosemary extract exhibits promising antiobesity effects (Zhao et al., 2015). To explore the mechanism of the antiobesity activity of rosemary, CA, carnosol, and rosmarinic acid were screened for antiobesity effects in HFD‐induced obese mice. The results demonstrated that carnosol and rosmarinic acid did not significantly affect body weight in mice, whereas CA significantly affected body weight gain after 10 weeks of feeding (*p* < .05, Table 2). In addition, CA did not decrease the weight of the spleen and liver but alleviated the weight gain of the kidney caused by the HFD. Moreover, HCA caused a significant reduction in food efficiency (*p* < .05) compared with that caused by the LCA and HFD groups.

**TABLE 2:** Body weights, food behaviors, and organ weights of mice under different treatments: HFD, HFD plus 0.1% carnosic acid (LCA), HFD plus 0.2% carnosic acid (HCA), HFD plus 0.2% carnosol (CO), HFD plus 0.2% rosmarinic acid (RA), and normal diet (ND)*

	HFD	LCA	HCA	CO	RA	ND
Initial weight (g)	19.527 ± 1.282^a^	19.641 ± 1.214^a^	19.331 ± 1.180^a^	19.309 ± 1.315^a^	19.458 ± 1.327^a^	19.356 ± 1.351^a^
Final weight (g)	32.640 ± 1.871^a^	27.890 ± 1.199^b^	25.298 ± 1.410^c^	30.479 ± 1.931^a^	31.827 ± 1.893^a^	26.589 ± 1.908^c^
Food intake (g)	2.015 ± 0.216^a^	1.973 ± 0.286^a^	2.056 ± 0.314^a^	2.022 ± 0.205 ^a^	1.986 ± 0.337^a^	2.213 ± 0.326^b^
Food efficiency^#^	0.087 ± 0.014^a^	0.059 ± 0.008^b^	0.041 ± 0.007^c^	0.072 ± 0.011^a^	0.081 ± 0.015^a^	0.046 ± 0.013^bc^
Fat (g)^※^	1.984 ± 0.413^a^	1.163 ± 0.326^b^	0.892 ± 0.085^c^	1.831 ± 0.361^a^	1.794 ± 0.305^a^	1.070 ± 0.191^c^
Liver (g)	1.15 ± 0.119^a^	1.04 ± 0.098^a^	0.974 ± 0.131^a^	1.137 ± 0.128^a^	0.982 ± 0.232^a^	0.981 ± 0.121^a^
Kidney (g)	0.382 ± 0.046^a^	0.356 ± 0.042^a^	0.318 ± 0.032^b^	0.372 ± 0.055^a^	0.363 ± 0.084^a^	0.325 ± 0.051^a^
Spleen (g)	0.102 ± 0.019^a^	0.106 ± 0.020^a^	0.105 ± 0.029^a^	0.103 ± 0.023^a^	0.110 ± 0.025^a^	0.113 ± 0.036^a^

* Data were expressed as the mean ± standard deviation and were analyzed using ANOVA followed by post hoc test. Different letters (i.e., a, b, and c) in superscript indicated the statistical significance level *p* < .05.

^#^ Food efficiency was calculated as gain of body weight/food intake.

^※^ Body fat was calculated by the sum of the epididymal, retroperitoneal, and mesenteric adipose tissues.

### 3.3 Carnosic acid modulated structural composition of the gut microbiota

CA exhibited significant antiobesity effects, but the relationship between the antiobesity activity of CA and its modulating effect on the gut microbiota has not yet been well investigated. Hence, 16S rRNA gene sequence analysis was performed.

The results of the alpha diversity analysis indicated that HFD feeding markedly reduced bacterial richness and diversity, as verified by the increased Simpson index and reduced Chao 1 and Shannon indices compared with those obtained for the ND group (Table 3). Nevertheless, HCA significantly ameliorated the diversity of gut microbes (*p* < .05).

**TABLE 3:** Shannon, Chao 1, and Simpson indexes of different group

	Shannon	Simpson	Chao 1
HFD	4.13 ± 0.33	0.94 ± 0.04	266.94 ± 62.87
HCA	5.74 ± 0.50*	0.75 ± 0.05*	510.46 ± 58.83*
LCA	5.46 ± 0.47	0.83 ± 0.02	409.34 ± 19.65
ND	6.07 ± 0.42*	0.82 ± 0.05*	620.44 ± 65.42*

* Data were expressed as the mean ± standard deviation (*n* = 6) and were analyzed using ANOVA followed by post hoc test. *p* < .05, compared with HFD group.

**FIGURE 2:**
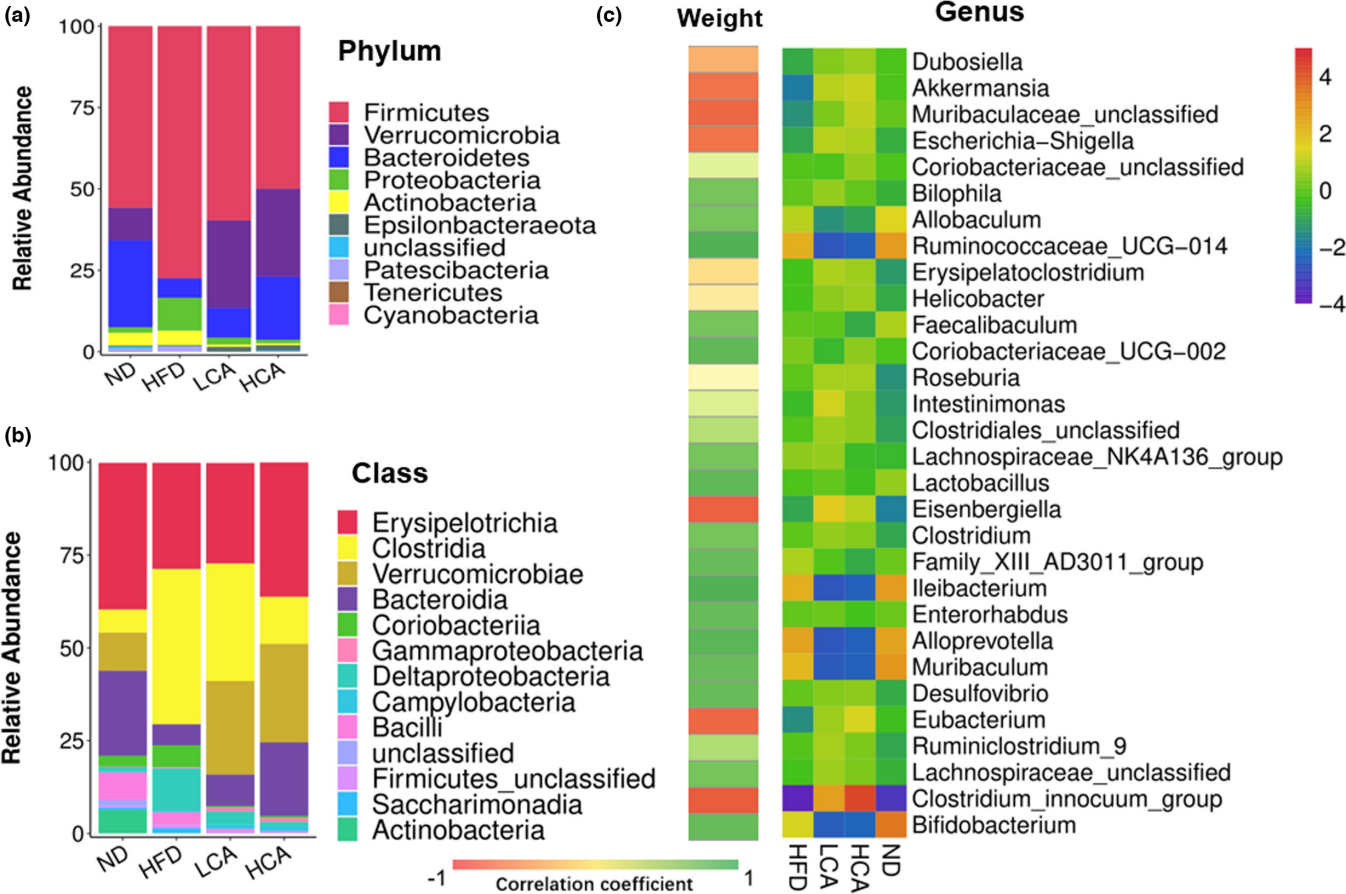
Effects of carnosic acid on the composition of fecal microbial in HFD‐fed C57BL/6 mice at (a) phylum, (b) class, and (c) genus (top 30) levels. The right heat map shows the average abundance values of top 30 microbial genera in each group which were processed with logarithmic normalization. The left heat map shows the Spearman correlations of the abundance of top 30 genera and the body weight among HCA and HFD groups. The *r* values are represented by gradient colors, where green and red cells indicate negative and positive correlations, respectively; *p < .05, **p < .01, compared with HFD group

At the phylum level, the microbial community was mainly composed of *Firmicutes*, *Verrucomicrobia*, *Bacteroidetes*, *Proteobacteria*, and *Actinobacteria* (Figure 2a). The HCA and LCA groups had a higher abundance of *Verrucomicrobia*, *Bacteroidetes*, and *Epsilonbacteraeota*, and a lower abundance of *Firmicutes*, *Proteobacteria*, and *Actinobacteria* compared with that of the HFD group (*p* < .05, Table S1). The ratios of *Firmicutes*/*Bacteroidetes* (F/B) for HFD, LCA, HCA, and ND were 13.22 ± 0.31, 7.08 ± 0.58, 2.42 ± 0.14, and 2.35 ± 0.50, respectively. The results indicated that the decrease in the F/B ratio caused by CA was dose dependent and that the HCA group could lower the F/B ratio to a level similar to that of the ND group.

The microbial community composition at the class level (relative abundance >0.3%) is shown in Figure 2b. In the LCA and HCA groups, *Verrucomicrobiae* were significantly more abundant (25.21% ± 2.84% and 26.52% ± 0.50%, respectively) than in the ND and HFD groups (10.26% ± 0.25% and 0.08% ± 0.02%, respectively). In addition, the HCA and LCA groups had a significantly higher abundance of *Gammaproteobacteria* and *Campylobacteria* and a significantly lower abundance of *Coriobacteriia*, *Bacilli*, *Saccharimonadia*, *Deltaproteobacteria*, and *Actinobacteria* than that of the HFD group (*p* < .05, Table S1). Compared with the HFD group (5.63% ± 0.38%), *Bacteroidia* increased in a dose‐dependent manner in the LCA and HCA groups (8.44% ± 0.76% and 20.26% ± 0.56%, respectively). In contrast, *Clostridia* decreased in a dose‐dependent manner in the LCA and HCA groups (31.66% ± 5.49% and 12.64% ± 6.08%, respectively) compared with that of the HFD group (41.72% ± 5.99%).

**FIGURE 3:**
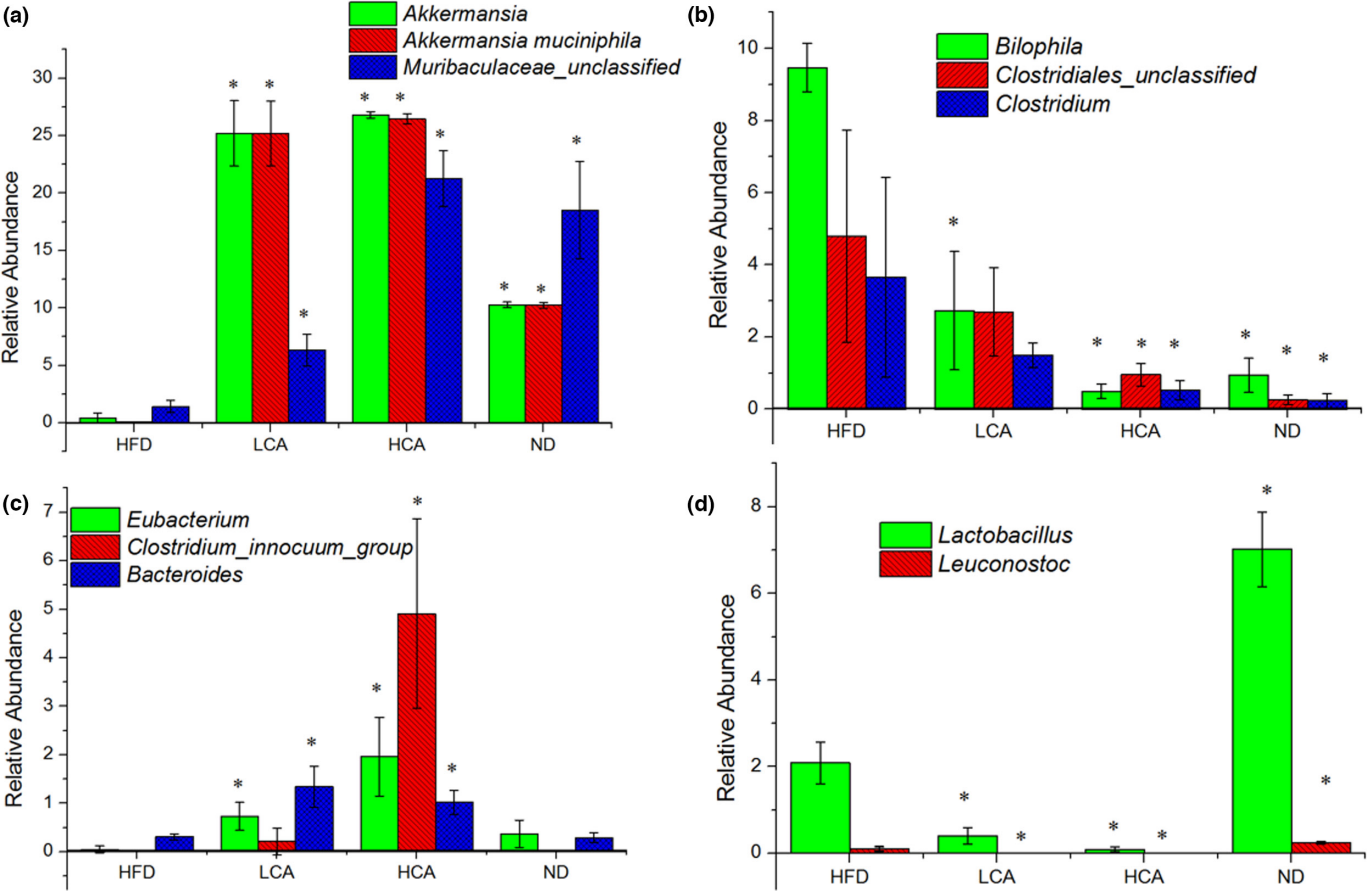
The relative abundance of *Muribaculaceae unclassified*, *Akkermansia*, *Akkermansia muciniphila* (a), *Bilophila* and *Clostridiales unclassified*, *Clostridium* (b), *Eubacterium*, *Clostridium innocuum* group, *Bacteroides* (c), *Lactobacillus*, and *Leuconostoc* (d), under different treatment. *p < .05, compared to HFD group

The abundances of the top 30 genera in each group are shown in Figure 2c. The bacterial genera exhibiting the most significant increase in relative abundance after CA treatment were *Akkermansia* and *Muribaculaceae unclassified*. CA increased the relative abundance of these genera to a similar or higher level than that in the ND group (Figure 3a). In contrast, CA decreased the relative abundance of *Bilophila*, *Clostridiales unclassified*, and *Clostridium* in a dose‐dependent manner (Figure 3b and Table S1). Moreover, the relative abundance of *Eubacterium* and *Clostridium innocuum* was significantly higher in the HCA group, while the relative abundances of *Allbaculum*, *Muribaculum*, *Ruminococcaceae UCG‐014*, *Coriobacteriaceae UCG‐002*, *Lachnospiraceae NK4A136* group, *Lactobacillus*, *Family XIII AD3011*, *Ileibacterium*, *Enterorhabdus*, *Muribaculum*, *Desulfovibrio*, *Ruminiclostridium 9*, and *Lachnospiraceae unclassified* were significantly lower in the CA treatment groups (*p* < .05, Table S1).

In addition, this study discovered that CA significantly increased the abundances of *Blautia* and *Bacteroides* and significantly decreased *Lactobacillus*, *Clostridium*, and *Leuconostoc* (*p* < .05, Figure 3c,d, and Table S1). This result is consistent with that reported in previous literature (Romo‐Vaquero et al., 2014).

*Akkermansia muciniphila* has been reported to exert an antiobesogenic effect and has been shown to significantly decrease obesity in animals. Owing to the notable bloom of *Akkermansia*, we compared the relative abundance of *Akkermansia muciniphila* among different groups. The results implied that HFD feeding significantly decreased the abundance of *Akkermansia muciniphila* compared with the effects of the normal diet, whereas *Akkermansia muciniphila* was significantly increased in the LCA and HCA groups (*p* < .05, Figure 3a and Table S1).

### 3.4 Identification of key bacterial genera associated with weight regulation

Spearman correlation analysis was performed to identify key genera that were potentially relevant to the bodyweight of mice in the HFD and HCA groups. Four genera were significantly associated with a decrease in weight, namely: *Akkermansia*, *Muribaculaceae unclassified*, *Escherichia–Shigella*, and the *Clostridium innocuum* group. (Figure 2c, *p* < .05, Table S2). The phylotypes that were significantly associated with an increase in weight were the genera *Allobaculum*, *Ruminococcaceae UCG‐014*, *Erysipelatoclostridium*, *Coriobacteriaceae UCG‐002*, *Roseburia*, *Lactobacillus*, *Eisenbergiella*, *Family XIII AD3011* group, *Ileibacterium, Enterorhabdus, Alloprevotella, Muribaculum, Desulfovibrio, Eubacterium, and Bifidobacterium*.

### 3.5 Differential taxa in different fecal microbial communities

**FIGURE 4:**
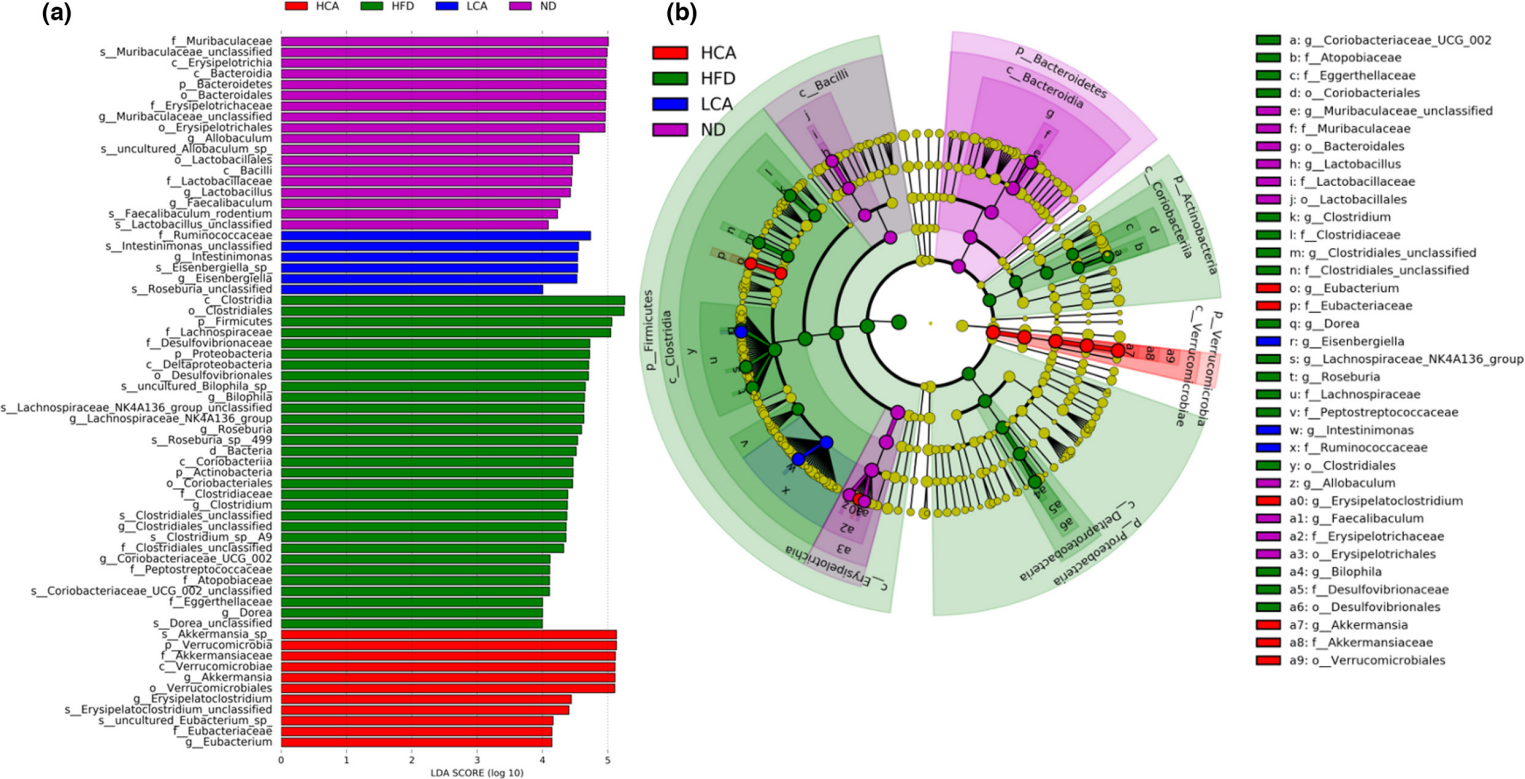
The linear discriminant analysis effect size (LEfSe) analysis of microbial abundance among different groups. (a) Histogram of the linear discriminant analysis (LDA) scores for differentially abundant bacteria taxa among different treatments (LDA threshold score was 4.0). (b) The cladogram of detected prokaryotic taxa for different groups

Linear discriminant analysis effect size (LEfSe) was performed to identify the enriched bacteria in each group. A total of 66 prokaryotic clades were screened out with an LDA threshold score of 4.0 (Figure 4a).

Taxa with significantly higher abundance in the ND group (Figure 4b) mainly belonged to the class *Bacteroidia* (including the genus *Muribaculaceae unclassified*, family *Muribaculaceae*, and order *Bacteroidales*), class *Bacilli* (including the genus *Lactobacillus*, family *Lactobacillaceae*, and order *Lactobacillales*), and class *Erysipelotrichia* (including the genera *Allobaculun* and *Faecalibaculum*, family *Erysipelotrichaceae*, and *Erysipelotrichales*).

The long‐term intake of an HFD instead of an ND increased the proportion of the classes *Coriobacteriia* (including the families *Atopobiaceae* and *Eggerthellaceae*, genus *Coriobacteriaceae UCG002*, and order *Coriobacteriales*), *Clostridia* (including the genus *Clostridium*, family *Clostrididiaceae*, genus *Clostridiales unclassified*, family *Clostridiales unclassified*, genus *Lachnospiraceae NK4A136* group, genus *Roseburia*, family *Lachnospiraceae*, family *Peptostreptococcaceae*, and order *Clostridiales*), and *Deltaproteobacteria* (including the *Akkermansia muciniphila*, genus *Bilophila*, family *Desulfovibrionaceae*, and order *Desulfovibrionales*). However, CA supplementation attenuated this microbial change in the HFD‐fed mice. Moreover, the relative abundance of the class *Verrucomicrobiae* (including *Akkermansia muciniphila*, genus *Akkermansia*, family *Akkermansiaceae*, and order *Verrucomicrobiales*) in the HCA group was significantly higher than that in the other groups. The genera *Eubacterium* and *Erysipelatoclostridium*, affiliated with the family *Eubacteriaceae*, were also enriched in the HCA group, whereas the genera *Eisenbergiella*, *Intestinimonas*, and *Ruminococcaceae* were enriched in the LCA group.

## 4 DISCUSSION

The dosages of CA were selected based on previous studies that reported that 0.28% rosemary extract (containing 80% CA) could ameliorate obesity induced by an HFD in mice (Zhao et al., 2015), and that 0.5% rosemary extract (containing 40% CA) significantly changed the microbiota composition of female Zucker rats (Romo‐Vaquero et al., 2014). Consistent with the literature, this study found that 0.1% CA significantly attenuated the body weight gain in mice.

It has been reported that the rosemary extracts (containing 40% CA) reduced the *Lactobacillus/Leuconostoc/Pediococccus* groups and increased the *Blautia coccoides* and *Bacteroides/Prevotella* in both lean and obese rats (Romo‐Vaquero et al., 2014). *Clostridium leptum* increased and *Bifidobacterium* decreased only in lean rats (Romo‐Vaquero et al., 2014). Our results confirmed a similar shift in the population of *Blautia*, *Bacteroides*, *Clostridium*, *Lactobacillus*, and *Leuconostoc*.

Previous studies have established that the obese microbiome in both humans and mice has an increased abundance of *Firmicutes* and *Proteobacteria* and a decreased *abundance of Bacteroidetes and Verrucomicrobia* (Ley et al., 2006; Turnbaugh et al., 2006). The gut microbiota of obese humans and animals exhibit a higher F/B ratio than that of normal weight individuals. Our results indicated that CA increased the abundance of *Verrucomicrobia* and *Bacteroidetes*. Moreover, CA reduced the abundance of *Proteobacteria* and the F/B ratio in a dose‐dependent manner. The reduced F/B ratio suggests that the changed microbial components might lead to a lower efficacy for energy harvesting. The reduced *Proteobacteria* levels suggested that CA could alleviate the microbial disorder caused by HFD, since the prevalence of *Proteobacteria* was recognized as a signature for microbial dysbiosis in the obese microbiome (Shin et al., 2015).

The interaction between bile acid and the gut microbiome is well‐known (al‐Sereiti et al., 1999). Previous reports state that the increase in *Bilophila* is associated with an HFD and bile acid metabolism (David et al., 2014). In addition, decreased intestinal bile acid‐related microbes, including *Lactobacillus*, *Lactococcus*, and *Clostridium*, increase the levels of ileal conjugated bile acids, which in turn inhibit the intestinal FXR‐FGF15 signaling pathway, resulting in reduced hepatic cholesterol and lipogenesis (Huang et al., 2019). In this study, CA reduced the levels of *Bilophila*, *Clostridium*, *Lactobacillus*, and *Leuconostoc*. Thus, inhibiting bile acid‐metabolizing bacteria could possibly be a pathway for the anti‐obesitogenic effect of CA.

The Spearman correlation analysis of the top 30 bacterial genera indicated that *Akkermansia*, *Muribaculaceae unclassified*, *Escherichia–Shigella*, and the *Clostridium innocuum* group were associated with a decrease in weight, while others were positively correlated with an increase in weight. Our results showed that CA supplementation significantly decreased the levels of positively correlated bacterial genera and increased the levels of negatively correlated bacterial genera.

The abundance of *Akkermansia muciniphila* in healthy individuals is higher than that in obese individuals, and it has been identified as beneficial bacteria (Everard et al., 2013). Administration of *Akkermansia muciniphila* could reverse metabolic disorders caused by HFD, such as insulin resistance, adipose tissue inflammation, and fat mass gain. The increase in *Akkermansia muciniphila* was the most significant in this study, suggesting that the prebiotic effect of *Akkermansia muciniphila* is crucial for the antiobesity activity of CA.

## 5 CONCLUSION

In conclusion, CA exhibits body weight–reducing effects and causes marked changes in the gut microbiota of HFD‐induced obese mice. The LEfSe indicated that CA attenuated the microbial changes caused by HFD. The modulating effect of CA on the gut microbiota was characterized by the promotion of probiotics and functional bacteria, including *Akkermansia muciniphila* and *Bacteroidetes*, and the inhibition of bile acid‐metabolizing bacteria, including *Bilophila*, *Clostridium*, *Lactobacillus*, and *Leuconostoc*. Thus, the promotion of probiotics and functional bacteria and the inhibition of bile acid‐metabolizing bacteria could be potential mechanisms by which rosemary elicits its antiobesity effects. Our results complement the current knowledge regarding the bioactivity of the phenolic constituents of rosemary.

## CONFLICT OF INTEREST

The authors declare that they have no conflict of interest.

## ETHICAL APPROVAL

This study was approved by the Ethics Committee for Animal Experimentation of the Wuyi University (Jiangmen, China).

## Supporting information

Tab S1‐S2

## Data Availability

The data that support the findings of this study are available from the corresponding author uponreasonable request.
