# Supplementary material for: Alteration of gut microbiota in high‐fat diet‐induced obese mice using carnosic acid from rosemary
Source: Food Sci Nutr. 2022 May 24;10(7):2325–32. doi: 10.1002/fsn3.2841 (PMC9281947; doi:10.1002/fsn3.2841)
Supplement: Supplementary file 1 — Tab S1‐S2 [file FSN3-10-2325-s001.docx]

**Supporting information for**

**Alteration of gut microbiota** **in high-fat diet-induced obese mice using carnosic acid from rosemary**

Xuan He^1^, Man Zhang^2^, Shu-Ting Li ^1^, Xinyu Li^1^, Qingrong Huang^1,2^, Kun Zhang ^1, 3^, Xi Zheng^1^, Xue-Tao Xu^1, 3^, Deng-Gao Zhao^1, 3, *^ , Yan-Yan Ma ^1, 3,*^

^1^School of Biotechnology and Health Sciences, Wuyi University, Jiangmen 529020, China.

^2^Department of Food Science, Rutgers University, 65 Dudley Road, New Brunswick, New Jersey, NJ 08901, USA.

^3^International Healthcare Innovation Institute (Jiangmen), Jiangmen 529040, China.

***corresponding authors**

E-mail: mayanyan@wyu.edu.cn, Tel/fax: +86-0750-3299391

List of content

| Page | Content |
| --- | --- |
| 3 | Table S1 |
| 6 | Table S2 |

**Table S1**. The relative abundance of key gut microbial taxa under different treatment. *

|  | Phylotypes | HFD | LCA | HCA | ND |
| --- | --- | --- | --- | --- | --- |
| Phylum | *Firmicutes* | 74.54±3.09^a^ | 59.80±0.88^b^ | 48.96±1.11^c^ | 53.70±2.05^d^ |
|  | *Verrucomicrobia* | 0.08±0.02 ^a^ | 25.21±2.84^b^ | 26.52±0.50^b^ | 10.26±0.25^c^ |
|  | *Bacteroidetes* | 5.63±0.38 ^a^ | 8.44±0.76 ^b^ | 20.26±0.76 ^c^ | 22.89±2.10^c^ |
|  | *Proteobacteria* | 11.75±1.80^a^ | 4.67±2.18^b^ | 1.74±0.46 ^b^ | 1.30±0.67 ^b^ |
|  | *Actinobacteria* | 6.06±1.83 ^a^ | 0.44±0.21 ^b^ | 0.55±0.36 ^b^ | 9.18±0.23^c^ |
|  | *Epsilonbacteraeota* | 0.45±0.14^a^ | 1.31±0.21^b^ | 1.76±0.08^c^ | 0.32±0.32^a^ |
| Class | *Erysipelotrichia* | 28.63±8.70^ab^ | 27.07±5.59^a^ | 36.22±6.99^ab^ | 39.52±1.83^b^ |
|  | *Clostridia* | 41.72±5.99^a^ | 31.66±5.49^a^ | 12.64±6.08^b^ | 6.23±2.09^b^ |
|  | *Verrucomicrobiae* | 0.08±0.02^a^ | 25.21±2.84^b^ | 26.52±0.50^b^ | 10.26±0.25^c^ |
|  | *Bacteroidia* | 5.63±0.38^a^ | 8.43±0.76 ^b^ | 19.92±0.56^c^ | 22.89±2.10^c^ |
|  | *Coriobacteriia* | 5.93±1.79^a^ | 0.42±0.18^b^ | 0.54±0.37^b^ | 2.88±0.27^c^ |
|  | *Gammaproteobacteria* | 0.34±0.04^a^ | 1.06±0.47^b^ | 1.05±0.14^b^ | 0.15±0.12^a^ |
|  | *Deltaproteobacteria* | 11.34±1.79^a^ | 3.41±2.13^b^ | 0.99±0.07^b^ | 1.15±0.55^b^ |
|  | *Campylobacteria* | 0.45±0.14^a^ | 1.30±0.17^b^ | 1.43±0.50^b^ | 0.32±0.32^a^ |
|  | *Bacilli* | 3.38±0.34^a^ | 0.48±0.15^b^ | 0.22±0.16^b^ | 7.40±0.83^c^ |
|  | *Saccharimonadia* | 1.32±0.32^a^ | 0.005±0.007^b^ | 0±0^b^ | 0.63±0.21^c^ |
|  | *Actinobacteria* | 0.13±0.04^a^ | 0.023±0.023^b^ | 0.009±0.009^b^ | 6.30±0.33^c^ |
| Genus | *Dubosiella* | 13.46±12.47^a^ | 22.40±8.18^a^ | 26.00±16.94^a^ | 26.94±3.19^a^ |
|  | *Akkermansia* | 0.42±0.43^a^ | 25.21±2.84^b^ | 26.81±0.27^b^ | 10.26±0.25^c^ |
|  | *Muribaculaceae_unclassified* | 1.42±0.52^a^ | 6.33±1.36^b^ | 21.26±2.46^c^ | 18.52±4.22^c^ |
|  | *Escherichia-Shigella* | 0.09±0.04^a^ | 0.73±0.36^b^ | 0.35±0.10^b^ | 0.07±0.06^a^ |
|  | *Coriobacteriaceae_unclassified* | 1.44±1.18^ab^ | 0.09±0.11^a^ | 0.40±0.17^b^ | 0.52±0.70^ab^ |
|  | *Bilophila* | 9.47±0.67^a^ | 2.73±1.64^b^ | 0.50±0.20^b^ | 0.94±0.47^b^ |
|  | *Allobaculum* | 4.31±0.92^a^ | 0.002±0.002^b^ | 0.002±0.003^b^ | 7.24±1.70^c^ |
|  | *Ruminococcaceae_UCG-014* | 1.02±0.24^a^ | 0±0^b^ | 0±0^b^ | 1.63±1.44^a^ |
|  | *Erysipelatoclostridium* | 3.21±4.38^ab^ | 4.24±2.98^ab^ | 1.73±0.58^a^ | 0.18±0.25^b^ |
|  | *Helicobacter* | 1.97±2.35^a^ | 1.30±0.17^a^ | 1.22±0.61^a^ | 0.32±0.32^a^ |
|  | *Faecalibaculum* | 1.07±0.76^a^ | 0.07±0.02^b^ | 0.0006±0.0007^b^ | 3.49±3.25^a^ |
|  | *Coriobacteriaceae_UCG-002* | 3.30±1.09^a^ | 0.03±0.03^b^ | 0.28±0.02^b^ | 0.48±0.15^b^ |
|  | *Roseburia9.47* | 5.78±7.90^a^ | 3.78±1.65^a^ | 2.13±1.39^a^ | 0.13±0.07^b^ |
|  | *Intestinimonas* | 1.63±1.86^a^ | 7.17±2.84^b^ | 0.99±0.96^a^ | 0.14±0.14^a^ |
|  | *Clostridiales_unclassified* | 4.80±2.94^a^ | 2.70±1.23^ab^ | 0.96±0.31^b^ | 0.26±0.13^b^ |
|  | *Lachnospiraceae_NK4A136_group* | 6.27±6.45^a^ | 0.79±0.99^b^ | 0.04±0.03^b^ | 0.26±0.17^b^ |
|  | *Lactobacillus* | 2.09±0.48^a^ | 0.40±0.19^b^ | 0.09±0.05^b^ | 7.02±0.86^c^ |
|  | *Eisenbergiella* | 0.19±0.12^a^ | 6.51±4.97^b^ | 0.88±0.38^a^ | 0.01±0.02^a^ |
|  | *Clostridium* | 3.63±2.77^a^ | 1.50±0.34^ab^ | 0.53±0.27^b^ | 0.24±0.20^b^ |
|  | *Family_XIII_AD3011_group* | 4.09±4.32^a^ | 0.04±0.04^b^ | 0.005±0.0007^b^ | 0.46±0.45^b^ |
|  | *Ileibacterium* | 0.97±0.37^a^ | 0±0^b^ | 0±0^b^ | 1.48±0.82^a^ |
|  | *Enterorhabdus* | 2.51±1.12^a^ | 0.29±0.21^b^ | 0.06±0.03^b^ | 1.64±0.81^a^ |
|  | *Alloprevotella* | 1.85±2.79^a^ | 0±0^b^ | 0±0^b^ | 0.91±1.35^a^ |
|  | *Muribaculum* | 0.65±0.34^a^ | 0±0^b^ | 0±0^b^ | 1.94±2.78^a^ |
|  | *Desulfovibrio* | 2.36±1.48^a^ | 0.48±0.37^b^ | 0.37±0.15^b^ | 0.14±0.08^b^ |
|  | *Eubacterium* | 0.05±0.07^a^ | 0.73±0.29^b^ | 1.96±0.81^b^ | 0.37±0.28^a^ |
|  | *Ruminiclostridium_9* | 1.73±1.68^a^ | 1.36±0.25^a^ | 0.29±0.16^b^ | 0.13±0.03^b^ |
|  | *Lachnospiraceae_unclassified* | 1.18±0.52^a^ | 1.29±0.73^a^ | 0.31±0.17^b^ | 0.29±0.26^b^ |
|  | *Clostridium_innocuum_group* | 0±0^a^ | 0.21±0.28^a^ | 4.91±1.95^b^ | 0±0^a^ |
|  | *Bifidobacterium* | 0.07±0.03^a^ | 0±0^b^ | 0±0^b^ | 6.16±0.23^c^ |
|  | *Leuconostoc* | 0.10±0.05^a^ | 0±0^b^ | 0±0^b^ | 0.24±0.03^c^ |
|  | *Lactococcus* | 0.36±0.01^a^ | 0±0^b^ | 0±0^b^ | 0.085±0.03^c^ |
|  | *Bacteroides* | 0.31± 0.06^a^ | 1.34± 0.42^b^ | 1.02 ± 0.25^b^ | 0.29 ± 0.10^a^ |
|  | *Blautia* | 0.02 ± 0.01 ^a^ | 0.04 ± 0.02^a^ | 0.26± 0.12 ^b^ | 0.01 ± 0.001^a^ |
| Specie | *Akkermansia muciniphila* | 0.08±0.02^a^ | 25.18±2.83 ^b^ | 26.47±0.45 ^b^ | 10.25±0.25 ^c^ |

*Data are expressed as the mean ± SD (n = 6) and were statistically analyzed using one-way ANOVA followed by Duncan’s multiple-range test. Different letters (i.e., a, b, c) in superscript indicated the statistical significance level p<0.05.

**Table S2.** Spearman correlation of relative abundance of top 30 bacterial genera with the body weight of HFD and HCA mice.

| genus | *r* value | *p* value |
| --- | --- | --- |
| *Dubosiella* | -0.5000 | 0.0977 |
| *Akkermansia* | -0.7483 | 0.0043 |
| *Muribaculaceae_unclassified* | -0.7858 | 0.0024 |
| *Escherichia-Shigella* | -0.7554 | 0.0036 |
| *Coriobacteriaceae_unclassified* | 0.2429 | 0.4469 |
| *Bilophila* | 0.7572 | 0.0046 |
| *Allobaculum* | 0.7261 | 0.0052 |
| *Ruminococcaceae_UCG-014* | 0.8425 | 0.0043 |
| *Erysipelatoclostridium* | -0.3143 | 0.3197 |
| *Helicobacter* | -0.2000 | 0.5330 |
| *Faecalibaculum* | 0.7429 | 0.0056 |
| *Coriobacteriaceae_UCG-002* | 0.8001 | 0.0017 |
| *Roseburia* | -0.0357 | 0.9122 |
| *Intestinimonas* | 0.3071 | 0.3314 |
| *Clostridiales_unclassified* | 0.4873 | 0.0959 |
| *Lachnospiraceae_NK4A136_group* | 0.7572 | 0.0042 |
| *Lactobacillus* | 0.8021 | 0.0017 |
| *Eisenbergiella* | -0.8001 | 0.0021 |
| *Clostridium* | 0.7378 | 0.0069 |
| *Family_XIII_AD3011_group* | 0.7623 | 0.0031 |
| *Ileibacterium* | 0.8519 | 0.0006 |
| *Enterorhabdus* | 0.7921 | 0.0047 |
| *Alloprevotella* | 0.8275 | 0.0008 |
| *Muribaculum* | 0.7810 | 0.0026 |
| *Desulfovibrio* | 0.7798 | 0.0031 |
| *Eubacterium* | -0.1857 | 0.5632 |
| *Ruminiclostridium_9* | 0.5286 | 0.0772 |
| *Lachnospiraceae_unclassified* | 0.7158 | 0.0082 |
| *Clostridium_innocuum_group* | -0.8208 | 0.0011 |
| *Bifidobacterium* | 0.7974 | 0.0019 |
